# Geoenvironmental evaluation of leachate and soil pollution potential of an open dumpsite

**DOI:** 10.1007/s10661-026-15037-2

**Published:** 2026-03-03

**Authors:** Folahan O. Ayodele, Bamitale D. Oluyemi‑Ayibiowu, Joseph K. Ogunjobi, Oluwapelumi O. Ojuri

**Affiliations:** 1 Department of Civil and Environmental Engineering, Federal University of Technology Akure, Akure, Nigeria; 2 Department of Civil Engineering, Federal Polytechnic Ado-Ekiti, Ado Ekiti, Nigeria; 3 Department of Chemistry, Federal University of Technology Akure, Akure, Nigeria; 4 Built Environment and Sustainable Technologies (BEST) Research Institute, Liverpool John Moores University, Liverpool, L3 3AF UK

**Keywords:** Dumpsite, Leachate, Heavy metal, Pollution, Ecological hazard

## Abstract

This study assessed leachate and soil contamination at the Igbatoro dumpsite, Akure, Nigeria. Leachate samples were analyzed for heavy metals, BOD, and other physicochemical parameters. Soil samples were collected at depths of 0.5, 1.0, and 1.5 m to evaluate twenty-five (25) parameters, including pH, TDS, DO, COD, and heavy metals. Statistical analyses, including two-way ANOVA and correlation analysis, were performed. Leachate Pollution Index (LPI), revised LPI (r-LPI), Geo-accumulation Index (Igeo), Metal Pollution Index (MPI), Pollution Load Index (PLI), Degree of Contamination (Cdeg), and Potential Ecological Risk Factor (RI) were used to evaluate contamination levels of Igbatoro dumpsite. Results showed that Leachate pH ranged from 5.44 to 6.45 (mean 6.01), and metals like As and Cu exceeded the Federal Environmental Protection Agency (FEPA) limits. Strong negative correlations were observed between pH and most parameters, while temperature showed positive correlations with nitrate and Ni. An LPI of 13.65 and r-LPI of 40.39 exceeded pollution thresholds, indicating significant contamination. Soil analysis revealed elevated metal concentrations compared to the control, with Cd showing the highest Igeo value (–0.60). Location 6 recorded the highest MPI values for Cu and Cr, while most heavy metals fell within pollution classes, reflecting severe contamination. The potential ecological risk factor (RI) value of 174.70 indicates moderate ecological risk, with Cd posing a particularly high risk (Eir = 98.34). Overall, the study highlights considerable environmental and public health risks, underscoring the urgent need for remediation and adoption of sustainable waste management practices.

**Supplementary Information:**

The online version contains supplementary material available at 10.1007/s10661-026-15037-2.

## Introduction

The generation of municipal solid waste (MSW) has surged due to industrialization, population growth, urbanization, migration, etc. (Alao et al., 2023; Rodrigues et al., 2025). Unsustainable management of solid waste is becoming a bigger problem for the environment around the world, especially in developing countries where people and the environment are at severe risk from bad waste disposal practices (Espinoza Pérez et al., 2021; Ferronato & Torretta, 2019; Ugwu et al., 2021). Open dumpsites, which are yards with little or no environmental control, are prevalent in most urban and rural areas of developing nations. This is due to inadequate MSW infrastructure to handle the generated MSW (Mushtaq et al., 2020). They significantly contribute to environmental pollution through the uncontrolled release of hazardous chemicals into the ecosystems. Open dumpsites have become a major environmental issue, mostly because of their ability to generate leachate, a sometimes-dangerous liquid produced from precipitation filtering through decomposing solid waste (Akinbile et al., 2021; Nyirenda & Mwamba, 2022).

Nigeria has been identified as maintaining the highest ranking among fifty of the largest active dumpsites globally, accounting for 12% of these sites from thirty different nations (Alao, 2023). Inappropriate control of leachate leads to its percolation into groundwater and soil, introducing heavy metals and other pollutants to the surroundings. Rainwater percolation, high moisture content, and physical and biochemical interactions contribute to landfill leachate production, and the leachate characteristics are influenced by a wide range of variables, including site hydrology, landfill age, precipitation intensity, and mainly waste composition (Bisht et al., 2022).

Leachate generation remains a significant issue for MSW disposal systems (Rajoo et al., 2020), including engineered landfills that are even designed to reduce the negative impacts of waste. Leachate consists of a complicated mix of organic and inorganic pollutants, and it is a major environmental pollution concern related to dumpsites. It releases dangerous compounds that impair human health and the environment. Leachate generated from solid waste sources, including domestic, industrial, and biomedical, adversely affects the quality of soil, groundwater, and surface water. Usually, leachate contains, among other pollutants, organic matter, ammonia, nitrates, chlorides, sulphates, and heavy metals. Heavy metal contamination is caused more by human activities (Ayobami, 2022; Olaniyan et al., 2024; Yang et al., 2023). Heavy metal contamination sources include manufacturing (industrial activities), solid waste disposal, and emissions from motor vehicles, among others (Akoto et al., 2016; Jilani & Rashid, 2019; Kolawole et al., 2023; Ojuri et al., 2018a, b). Heavy metal contamination is one of the most persistent and hazardous consequences of open dumpsites (Dutta et al., 2022; El Fadili et al., 2022), and unlike organic pollutants that degrade over time, heavy metals are non-biodegradable and can accumulate in soil, plants, and living organisms through bioaccumulation and bio-magnification. The presence of toxic metals such as lead, cadmium, and mercury in soil and groundwater can significantly affect agricultural productivity, as plants grown in contaminated soil may absorb these metals, ultimately entering the food chain and threatening public health.

Additionally, the mobility and bioavailability of heavy metals in the environment depend on various factors, including pH, soil composition, and the presence of organic matter. Acidic conditions, for instance, enhance the solubility of metals, increasing their potential to leach into groundwater. The Igbatoro dumpsite, situated in a tropical region with high rainfall, may be particularly susceptible to this process, making it a high-hazard area for metal contamination.

It is important to have an environmental assessment of the Igbatoro dumpsite (the largest dumpsite in Akure, Nigeria) to understand the current conditions necessary to interpret various pollutant concentrations and heavy metal contamination of the dumpsite. This environmental hazard assessment is essential in the development of effective waste management and remediation strategies. However, there have been growing concerns about the grave consequences of open dumpsites, even the Igbatoro dumpsite. There is a lack of comprehensive studies assessing the environmental hazard posed by the Igbatoro dumpsite. This study aims to fill that gap by examining the physicochemical properties of leachate and the heavy metal content in the surrounding soil to evaluate the potential health and environmental impacts of uncontrolled dumping activities. The findings will offer critical insights into the hazards associated with the Igbatoro site, contribute to the broader understanding of open dumpsite pollution, and support environmental agencies and policymakers in developing targeted remediation strategies to promote sustainable solid waste management.

## Materials and methods

### Description of site

**Fig. 1:**
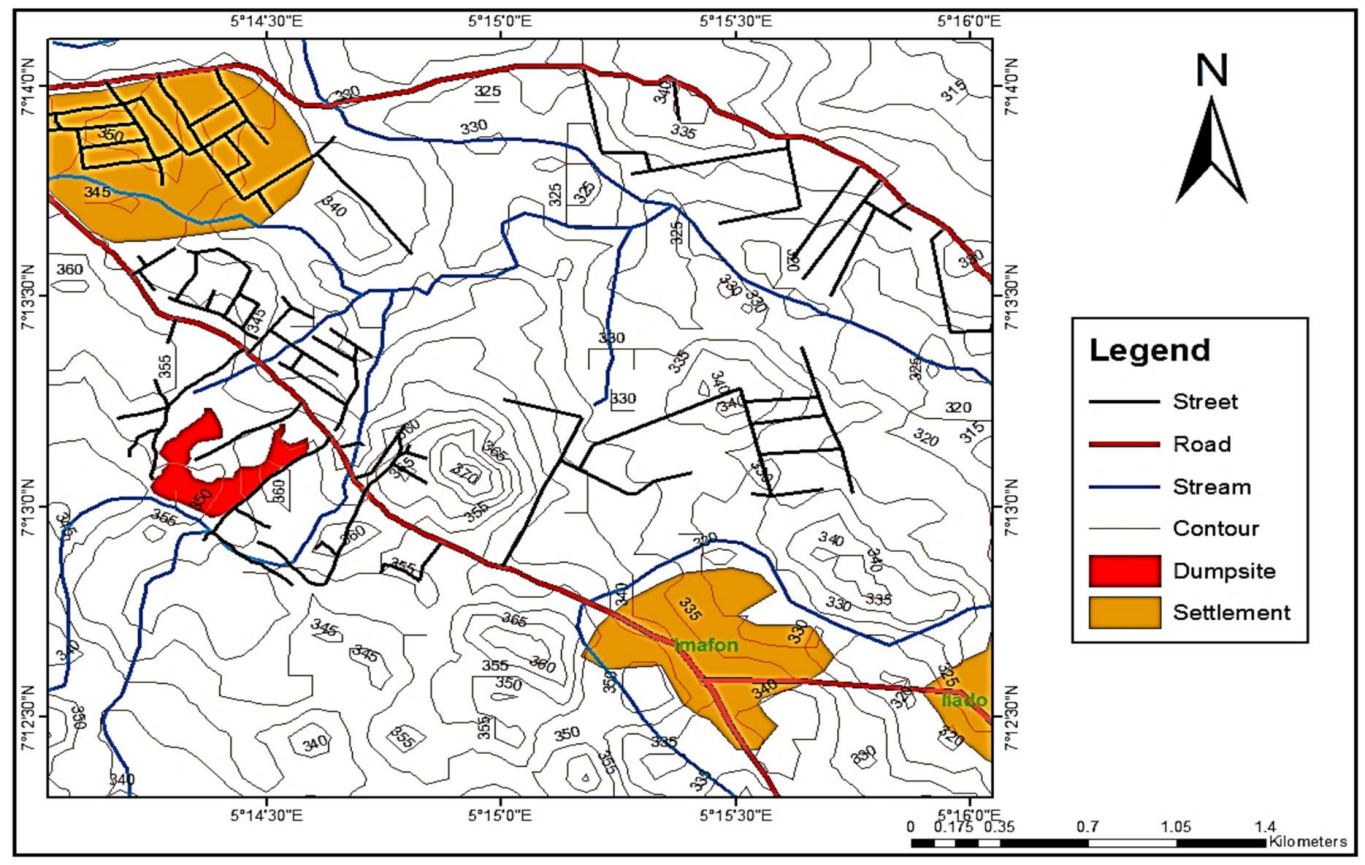
Map showing the study area and dumpsite location in Ondo State, Nigeria (Source: Ojuri et al., 2018a, b; Ayodele & Alo, 2020)

The shape file map of the study area (yard) presented in Fig. 1 is the Igbatoro dumpsite in Akure, the capital of Ondo State in Nigeria. Akure is a millennium city with a tropical humid climate, having its annual rainfall ranging between 1405 and 2400 mm on average (Ojuri et al., 2018a, b). The Igbatoro dumpsite is the largest and most active in Akure, Ondo State. The yard owned by the Ondo State Government functions as an open yet semi-controlled dumping site, characterised by perimeter fencing at the front elevation. The dumpsite occupies over six (6) hectares and receives both municipal solid waste and liquid waste, with an annual intake exceeding 100,000 metric tonnes (Ayodele et al., 2025; Elemile et al., 2019). The dumpsite is bordered by residential, commercial, and industrial structures, with Imafon (the closest village) being about 1.6 km upwind. The dumpsite is characterized by a tropical humid climate with two distinct seasons: harmattan and rainy. The average annual rainfall is between 1405 and 2400 mm. The parent material of the dumpsite’s soil is characterized by crystalline basement complex rocks, made up of ferruginous tropical soils. The measurements of some receptors of the dumpsite are shown in Table 1.

**Table 1:** Some receptors of the Igbatoro dumpsite (Source: Ojuri et al., 2018a, b)

S/N	Some critical receptors of the Igbatoro dumpsite	Attribute measurement
1	Distance from nearest water supply source (m)	4010
2	Distance to critical habitats such as wetlands and reserved forest (km)	25.9
3	Distance to the nearest airport (km)	7.2
4	Distance from surface water body (m)	< 500
5	Distance to the nearest village in the predominant wind (m)	1600
6	Flood proneness (flood period in years)	0
7	Distance from the city centre (km)	7.6
8	Groundwater depth (m)	7.2

### Materials sampling

#### Leachate sampling and testing methods

McCartney, Amber vial, and plastic bottles wrapped with fossil paper were used to sample the leachates from the dumpsite. McCartney bottles were used to collect leachate samples for the analysis of heavy metals, Amber sample vial bottles were utilised to collect leachate for the assessment of Biological Oxygen Demand (BOD_5_), whereas plastic bottles were employed for the collection of samples necessary for additional leachate parameters analysis. The dumpsite lacks a leachate pond; consequently, five (5) leachate samples were collected at random from a shallow pit excavated using a digger and shovel at locations lower in relation to the sampling area. Their coordinates are presented in Table 2.

**Table 2:** Coordinates of sampled leachate

Sample name	Latitude	Longitude	Elevation
IDLS1	7°13′186’’	5°14′463’’	353.8 m
IDLS2	7°13′159’’	5°14′499’’	357.6 m
IDLS3	7°13′126’’	5°14′495’’	360.2 m
IDLS4	7°13′093’’	5°14′493’’	359. 1 m
IDLS5	7°13′201’’	5°14′563’’	359. 6 m

The samples were kept in a cooler and transported to the lab for analysis. The parameters tested include temperature, Total dissolved solids (TDS), Chemical Oxygen Demand (COD), Dissolved Oxygen (DO), Biochemical Oxygen Demand (BOD), Chlorides, Ammonia, Total Nitrogen, Sulphate, Phosphate, Nitrate, Total Kjeldahl Nitrogen (TKN), Fecal Coliform Bacteria (FCB), Total Coliforms, Phenol, pH, Ammonia Nitrogen, heavy metals including Arsenic (As), Chromium (Cr), Copper(Cu), Cyanide(Cy), Iron (Fe), Lead (Pb), Manganese (Mn), Nickel (Ni), and Zinc (Zn). Temperature and pH were measured following ASTM D1293-99 (2005). TDS was determined and calculated from Eq. (1)

1 $$\mathrm{TDS}\;(\mathrm{mg}/\mathrm{l})=\frac{\left({\mathrm{W}}_{2}-{\mathrm{W}}_{1}\right) \times 1000}{100}$$

100 ml of the leachate sample was measured and filtered into an evaporating dish. They were dried and weighed as W_1_. The filtrates were transferred into a pre-weighed evaporating dish. Then they were heated to dryness at 105 °C. The process of drying, cooling, and weighing was continued until a constant weight (W_2_) was obtained.

An oxygen meter (DO-5510HA) was used to determine DO, while titrimetric and incubation methods following APHA (1999) were used to determine the BOD and COD. Chlorides, nitrate, sulphate, and phosphate were determined using titration methods following APHA (1999).To determine ammonia nitrogen, 1 ml of leachate was converted to ammonium sulphate through digestion with concentrated H_2_SO_4_ in the presence of CuSO_4_ and Na_2_SO_4_. Upon heating, the ammonia produced was steam-distilled into a boric acid solution. The nitrogen content from ammonia was determined through the titration of the captured ammonia using 0.1 M HCL and a Tashirus indicator (double indicator) until a purplish-pink colour was achieved. The determination of the heavy metals was done using an Atomic absorption spectrophotometer, AAS (Model: Buck Scientific 211VGP), after the leachate was filtered and digested in a 250 ml water sample with 10 ml of concentrated analytical grade nitric acid.

The process of determining phenol (C_6_H_6_O) required required the measurement of 0.5 g of the compound, which was then dissolved in a sufficient volume of water to reach a final volume of 500 ml in a volumetric flask. 25.0 ml of this solution was mixed with 25.0 ml of 0.1 N potassium bromate in a 250 ml iodine flask. Following this, 1 g of powdered potassium iodide (KI) and 10.0 ml of dilute hydrochloric acid were added to the mixture. The glass stopper was moistened with a few drops of KI solution and placed appropriately. The materials were subjected to a low-light setting for 20 min, accompanied by regular stirring during this time. 10 ml of KI solution was added, and the mixture was shaken thoroughly before being allowed to sit in the dark for an additional 5 min. The flask's stopper and neck underwent a thorough washing with distilled water, after which 10 ml of chloroform was added. The liberated iodine was subsequently titrated with 0.1 N sodium thiosulphate, utilising freshly prepared starch as an indicator. A blank titration was performed concurrently, and appropriate corrections were made as required. 1 ml of 0.1 N potassium bromate is equivalent to 0.001569 g of C_6_H_6_O. The Most Probable Number (MPN) enables the determination of coliform detection. It requires both the confirmatory and presumptive phases. Following manufacturer guidelines, MacConkey broth was made, and 25 ml of the broth was placed into a test tube. The tubes were filled with inverted vials, covered with aluminium foil, and cotton wool-plugged. It was sterilized for 15 min at 121℃ in an autoclave. The samples underwent serial dilution; the last three sets of dilutions were inoculated with 1 ml of the inoculum; each tube was plugged with cotton wool and incubated at 37 ℃ for 24 h. After twenty-four to forty-eight hours, fermentation and gas generation were noted. Non-fermented tubes were taken as negative; the tubes where fermentation or formation of gas happened were taken as positive. A confirmatory test was performed on the positive tubes. Every tube was sub-cultured into a fresh MacConkey broth medium; a sterile wire loop was used to place a loop full of culture into the fresh broth and plugged with cotton wool, then incubated at 37 ℃ for 24–48 h. The most probable number table enabled the determination of the density of coliform bacteria; the result was noted as (MPN/100 ml). These parameters were analyzed to assess the contamination potential of the dumpsite using the Leachate Pollution Index (LPI) and Revised Leachate Pollution Index (r-LPI).

#### Soil sampling and testing methods

The dumpsite's underlying soil samples were collected at 0.5, 1.0, and 1.5 m depth manually using a digger and shovel. The control sample was taken at a point more than 70 m away from the dumpsite. Table 3 presents the coordinates of the soil samples. The chemical parameters (calcium (Ca), sodium (Na), Magnesium (Mg), Potassium (K), and heavy metals) of the underlying soil of the dumpsite were assessed. The heavy metals tested include Chromium (Cr), Cadmium (Cd), Copper (Cu), Cobalt (Co), Manganese (Mn), Iron (Fe), Lead (Pb), Nickel (Ni), and Zinc (Zn). The soil samples were initially digested using a Kjeldahl flask with an aqua mixture, and the filtrate was then analyzed for heavy metals using AAS.

**Table 3:** Coordinates of sampled soil

Sample name	Latitude	Longitude	Elevation
IDSS1	$7^\circ {13{\prime}}160{\prime}{\prime}$	$5^\circ 14{\prime}463{\prime}{\prime}$	$369.1 m$
IDSS2	$7^\circ 13{\prime}141{\prime}{\prime}$	$5^\circ 14{\prime}474{\prime}{\prime}$	$374.0 m$
IDSS3	$7^\circ 13{\prime}109{\prime}{\prime}$	$5^\circ {14}{\prime}429{\prime}{\prime}$	$368.8 m$
IDSS4	$7^\circ 13{\prime}072{\prime}{\prime}$	$5^\circ 14{\prime}453{\prime}{\prime}$	$369.8 m$
IDSS5	$7^\circ 13{\prime}027{\prime}{\prime}$	$5^\circ {14}{\prime}418{\prime}{\prime}$	$362.8 m$
IDSS6	$7^\circ 13{\prime}089{\prime}{\prime}$	$5^\circ {14}{\prime}394{\prime}{\prime}$	$365.1 m$
IDSS7	$7^\circ 13{\prime}169{\prime}{\prime}$	$5^\circ 14{\prime}422{\prime}{\prime}$	$365.9 m$
IDSS8	$7^\circ 13{\prime}233{\prime}{\prime}$	$5^\circ 14{\prime}617{\prime}{\prime}$	$355.0 m$

### Statistical analysis

The assessment of the effect of location and depth on the chemical parameters of the soil was done by ANOVA (Two-way without replication) individually. The ANOVA was performed to measure the significance of the heavy metals in relation to depths and location on the dumpsite. Also, the correlation analysis of the leachate was assessed.

### Leachate pollution potential of the dumpsite

#### Leachate pollutant index (LPI) of igbatoro dumpsite

LPI is an increasing scale index, formulated based on the Delphi technique and a veritable tool for determining the contamination of waste disposal, especially in developing countries. There are eighteen (18) chemical parameters used for the LPI evaluation. Also, the parameters are subdivided into Organic (LPI_or_), Inorganic (LPI_in_), and heavy metals (LPI_hm_) with varying allotted weight factors ranging from 0.088 to 0.267 for the calculation of the overall LPI. Each parameter (pollutant) concentration obtained in the laboratory is used on the sub-index average curves provided by Kumar and Alappat (2005a) and Kumar and Alappat (2005b) to determine the sub-index value (p_i_), which is then multiplied by the allotted weight factor (w_i_). LPI is calculated using Eqs. 2 or 3. Equation 3 is used when the data for all the chemical parameters are not determined. Hence, this was used for this analysis.

2 $$LPI= \sum\nolimits_{i=1}^{n}{w}_{i}{p}_{i}$$

3 $$LPI= \frac{\sum_{i=1}^{m}{w}_{i}{p}_{i}}{\sum_{i=1}^{m}{w}_{i}}$$

The overall LPI is determined using Eq. 4

4 $$Overall \;LPI=0.232{LPI}_{or}+0.257{LPI}_{in}+0.511{LPI}_{hm}$$

LPI is the weighted additive leachate pollution index; wi is the ith pollutant variable weight; pi is the sub-index of the ith leachate pollutant variable; n is the total number of leachate pollutant variables applied for the analysis; while m is the count of available leachate pollution parameters.

#### Revised –leachate pollution index

The revised LPI (r-LPI) was developed in 2022 by Bisht et al. (2022) following the identified deficiencies of the old LPI developed by Kumar and Alappat (2005a, b). Two (2) extra parameters, such as pesticides and Faecal Coliform Bacteria (FCB), were incorporated in the computation of r-LPI, even though the r-LPI and the LPI had nine (9) similar chemical parameters. Toxicants had the highest weightage in the r-LPI because they are harmful to human health and the environment, while basic pollutants have the least weight as they are relatively less harmful to human health or the ecosystems. Heavy metals have moderate weightage. The heavy metals included in r-LPI are lead, arsenic, mercury, and chromium. Since they are quite poisonous, carcinogenic, and do not break down, these heavy metals seriously endanger human health as well as the environment. This offers a comprehensive tool for leachate pollution risk assessment.

Similar to the LPI and overall LPI, each parameter (pollutant) concentration obtained in the laboratory is used on the sub-index average curves provided by Bisht et al. (2022) to determine the sub-index value (p_i_), which is then multiplied by the allotted weight factor (w_i_). The weightage factors allotted to determine LPI, Overall LPI, and r-LPI are presented in Table 4.

**Table 4:** The allotted pollutant weight factor for LPI, Overall LPI, and r-LPI analysis

S/N	Parameters	LPI weight	Overall LPI weight factor (w_i_)	Overall r-LPI weight factor
1	pH	0.055	0.214	0.176
2	TDS	0.050	0.195	-
3	BOD	0.061	0.263	0.240
4	COD	0.062	0.267	0.278
5	TKN	0.053	0.206	-
6	Ammonia Nitrogen	0.051	0.198	-
7	Fe	0.045	0.088	-
8	Cu	0.050	0.098	-
9	Ni	0.052	0.102	-
10	Zn	0.056	0.110	-
11	Pb	0.063	0.123	0.255
12	Cr	0.064	0.125	0.140
13	Hg	0.062	0.121	0.374
14	As	0.061	0.119	0.231
15	Phenol	0.057	0.246	0.251
16	Chloride	0.048	0.187	-
17	Cy	0.058	0.114	0.451
18	Total coliform	0.052	0.224	-
19	FCB	-	-	0.305
20	Pesticides	-	-	0.299

Just like the LPI procedures, each parameter (pollutant) concentration obtained in the laboratory is used on the normalized curves to determine the normalized value of each parameter (p_i_), which is then multiplied by the allotted corresponding weight (w_i_). r-LPI is calculated using Eq. 5.

5 $$r- LPI= \frac{\sum_{i=1}^{n}{w}_{i}{p}_{i}}{\sum_{i=1}^{n}{w}_{i}}$$

where p_i_ is the normalized value of the parameters, and w_i_ is the corresponding weights.

The overall r-LPI is determined using Eq. 6

6 $$Overall \;LPI=0.380{LPI}_{Tox}+0.363{LPI}_{Hm}+0.257{LPI}_{Bp}$$

where LPI is the weighted additive leachate pollution index,

LPI_Tox_ is the Sub-LPI for the toxicants; LPI_Hm_ is the Sub-LPI for the heavy metals; and LPI_Bp_ is the Sub-LPI for the basic pollutants.

### Pollution indices of igbatoro dumpsite soil

#### Geoaccumulation index, I_geo_

The index of geoaccumulation (I_geo_) is a good soil contamination assessment tool developed by Muller in 1969. It is one of the quantitative indices used in the assessment of pollution levels (Oguntuase et al., 2024). It can be used to determine metal contamination in soil. It is calculated using Eq. 7.

7 $${\mathrm{I}}_{\mathrm{geo}}= {\mathrm{Log}}_{2 }\left[\frac{{\mathrm{C}}_{\mathrm{n}}}{1.5 {\mathrm{B}}_{\mathrm{n}}}\right]$$

where C_n_ is the concentration of a target metal.

1.5 is a constant used because of variations of a given metal and anthropogenic factors

B_n_ is the geochemical reference value of a target metal

The geochemical reference values used are 47,200 for Fe, 850 for Mn, 90 for Cr, 20 for Pb, 68 for Ni, 95 for Zn, 45 for Cu, and 0.30 for Cd as reported by Turekian & Wedepohl (1961) and used by Ihedioha et al. (2017). These values were adopted because of the unavailability of the geochemical background values for these heavy metals.

The classes of I_geo_ include I_geo_ $<$ 0 for unpolluted, 0 −1 for unpolluted to moderately polluted, 1–2 for moderately polluted, 2–3 for moderately to strongly polluted, 3–4 for strongly polluted, and 4—5 for strongly to extremely polluted (Aralu et al., 2024; Oguntuase et al., 2024; Olanipekun et al., 2024).

#### Metal pollution index (MPI), Pollution load index (PLI), and Degree of contamination

This indicates the relationship between metal in the studied soil and the control soil. It can be determined using Eq. 8.

8 $$\mathrm{MPI}= \frac{\text{Concentration of metal in the studied soil}}{\text{Control soil}}$$

MPI of 0.76–1.00 indicates very severe contamination, MPI of 0.51–0.75 indicates severe contamination, MPI of 0.26–0.5 indicates moderate contamination, and MPI of 0.1–0.25 indicates slight contamination. When MPI is $<$ 0.1, it indicates very slight contamination. All the contamination classes pose no risk to the soil, plants, and environment. However, for all MPI classes ranging from 1.1 to ≥ 16.0, it poses a negative effect. The 1.1–2.0 range indicates slight pollution, the 2.1–4.0 range indicates moderate pollution, the 4.1–8.0 range indicates severe pollution, the 8.1–16.0 range indicates very severe pollution, and $\ge$ 16.0 indicates excessive pollution. These values, classes, and their implication are stated by Ebong et al. (2019).

PLI also enables the estimation of pollution levels in the soil. It is calculated using Eq. 9

9 $$\mathrm{PLI}={{(\mathrm{MPI}}_{1}\times {\mathrm{MPI}}_{2 }\times \dots \dots {\mathrm{MPI}}_{\mathrm{n}})}^{{~}^{1}\!\left/ \!{~}_{n}\right.}$$

where n is the number of target heavy metals.

The PLI $<$ 0, 1 $<$ PLI $<$ 2, 2 $<$ PLI $<$ 3, PLI $>$ 3 indicate low-level, moderate-level, high-level, and extremely high-level pollution, respectively.

The summation of the MPI of the studied heavy metals ($\sum MPI$) is used to calculate the degree of contamination (C_deg_). The classification of C_deg_ proposed by Hakanson (1980) puts C_deg_ $<$ 8 for low contamination, from 8–16 for moderate contamination, C_deg_ from 16–32 for considerable contamination, and C_deg_ $\ge$ 32 for high contamination.

#### Ecological risk factor (E^i^r)and potential ecological risk factor (RI)

It was also proposed by Hakanson (1980). It is used to determine the potential ecological risk of target metal in the dumpsite soil, and the formula is presented in Eq. 10.

10 $${\mathrm{Er}}^{\mathrm{i}}= {\mathrm{T}}_{\mathrm{r}}\times \mathrm{MPI}$$

where T_r (_toxic response factor) is given as 5 for Ni, 0 for Fe, 5 for Cu, 30 for Cd, and 5 for Pb.

The RI is estimated by the addition of several heavy metal ecological risk factors.

If ${\mathrm{E}}^{\mathrm{i}}\text{r }<40$, the heavy metal load poses a low ecological risk, if ${40<E}^{\mathrm{i}}\text{r }\le 80$, the risk is moderate, and if ${80<E}^{\mathrm{i}}\text{r }\le 160$, the risk is appreciable. For high and serious ecological risk, ${160<E}^{\mathrm{i}}\text{r }\le 320$ and ${\mathrm{E}}^{\mathrm{i}}\text{r }>160,$ respectively. The RI class is as follows: $\mathrm{RI}<150$ for low ecological risk, $150<RI<300$ for moderate ecological risk, $300<RI<600$ for high ecological risk, and $\mathrm{RI}\ge 600$. These classes were reported by Ebong et al. (2019)

## Results and discussion

### Leachate characteristics of Igbatoro dumpsite

A total of twenty-five (25) parameters were analyzed and shown in Table 5. It was observed that the pH values of the leachate range from 5.44 to 6.45 and have a mean concentration of 6.01 ± 0.35. The pH value of 6.01 ± 0.35, which is a low value, is typical of pH values in developing countries like Nigeria due to dilution (Rajoo et al., 2020). The temperature of the sample varies from 25.35 to 26.65 $^\circ{\rm C}$ with a mean concentration of 25.84 ± 0.44.

**Table 5:** Leachate parameters and their concentration

S/N	Parameters	Min	Max	Mean ± SD	CV (%)	FEPA (1991) limits	Standard Limits (Jolaosho et al. 2024)	Indian leachate disposal standards (Agbozu & Oghama, 2015)
1	pH	5.44	6.45	6.01 ± 0.35	5.75	6—9	6—9	5.5–9.0
2	Temp	25.35	26.65	25.84 ± 0.44	1.70	-	-	-
3	TDS (mg/l)	215.97	506.23	393.60 ± 102.95	26.16	2000	1500	2100
4	T. Nitrogen (mg/l)	36.53	92.74	63.94 ± 20.85	32.61	-	-	-
5	TKN (mg/l)	40.48	110.24	74.25 ± 24.83	33.44	-	-	-
6	Ammonia Nitrogen (mg/l)	3.96	17.51	10.31 ± 4.35	42.16	-	-	-
7	Phenol (mg/l)	0.15	0.92	0.50 ± 0.26	51.78	-	-	-
8	Chloride (mg/l)	24.57	68.15	49.42 ± 14.80	29.94	-	-	1000
9	Sulphate (mg/l)	2.93	13.25	7.55 ± 3.71	49.15	-	-	NS
10	Ammonia (mg/l)	1.75	9.17	5.34 ± 2.43	45.53	-	0.01	-
11	Phosphate (mg/l)	0.98	8.31	4.80 ± 2.38	49.57	-	-	-
12	Nitrate (mg/l)	2.81	12.39	6.67 ± 3.17	47.45	-	-	-
13	BOD (mg/l)	8.76	12.54	11.05 ± 1.30	11.75	-	-	30
14	COD (mg/l)	14.27	18.48	16.40 ± 1.39	8.45	-	-	250
15	Total Coliform (MPN/100 ml)	15	95	58.00 ± 27.87	48.05	-	-	-
16	FCB (CFU/ml)	10	37	22.00 ± 8.82	40.10	-	-	-
17	As (mg/l)	0.06	0.24	0.16 ± 0.06	37.09	0.05	-	-
18	Cu (mg/l)	0.97	3.15	2.10 ± 0.70	33.36	0.2	$<$ 1	3.0
19	Cr (mg/l)	0.43	1.81	1.19 ± 0.46	38.70	0.05	$<$ 1	2.0
20	Cy (mg/l)	1.38	9.23	5.65 ± 2.53	44.74	-	-	-
21	Fe (mg/l)	5.09	24.15	14.54 ± 6.12	42.08	5	20	-
22	Mn (mg/l)	0.31	1.21	0.69 ± 0.30	44.23	0.2	0.05	-
23	Ni (mg/l)	0.08	0.38	0.18 ± 0.11	61.78	0.2	$<$ 1	-
24	Pb (mg/l)	0.32	1.92	1.15 ± 0.58	50.71	0.1	$<$ 1	0.1
25	Zn (mg/l)	5.69	16.24	10.57 ± 3.58	33.86	2	$<$ 1	5.0

The TDS (a vital parameter in assessing the discharge of leachate to the surrounding environment) has a mean concentration of 393.60 ± 102.95, ranging from 215.97 to 506.23 mg/l. The total nitrogen of the leachate ranged from 36.52 to 75.16, and the mean concentration was 63.94 ± 20.85. The mean TKN concentration (the addition of T. Nitrogen and Ammonia Nitrogen) of the dumpsite has an average value of 74.25 ± 24.83, while it varies from 40.48 mg/l to 110.24 mg/l. Ammonia nitrogen concentration varies from 3.96 mg/l to 17.51 mg/l with a mean concentration of 10.31 ± 4.35.

The Phenol concentration varies from 0.15 mg/l to 0.92 mg/l with a mean concentration of 0.50 ± 0.26. The phenolic compound (Phenol) concentration of the dumpsite is 0.50 ± 0.26, which is characteristically low, especially for African countries that predominantly dispose of waste into open dumping. The low value can be attributed to the degradation of the MSW associated with aerobic conditions (Abunama et al., 2021).

The chloride value of the leachate sample ranged from 24.57 to 68.15 with a mean concentration of 49.42 ± 14.80. Chloride is also a strong contamination indicator, which is highly mobile. The sulphate value of the leachate ranged from 2.93 to 13.25 and had a mean concentration of 7.55 ± 3.71. The Ammonia concentration, which can be used to assess the polluting potential, has a range of 1.74 mg/l to 9.169 mg/l and a mean concentration of 5.34 ± 2.43. Phosphate concentrations vary from 0.98 to 8.31 with a mean concentration of 4.80 ± 2.38. The nitrate has a range of 2.81 mg/l to 12.39 mg/l and a mean concentration of 6.67 ± 3.17.

The concentration varies from 8.76 mg/l to 12.54 mg/l and from 14.27 mg/l to 17.33 mg/l for BOD and COD, respectively. Their mean concentrations are 11.05 ± 1.30 and 16.40 ± 1.39, respectively. The BOD/COD ratio of the dumpsite is 0.67, which is greater than 0.4–0.6 or more, indicating that the leachate is young, having organic matter that is actively decomposing (Abunama et al., 2021). The total coliform concentration ranges from 15 to 95 MPN/100 ml, with a mean concentration of 58.00 ± 27.87, while FCB varies from 10to 37 CFU/ml, with a mean concentration of 22.00 ± 8.82.

For the heavy metal, the ranges and mean concentrations are 0.06 to 0.24 and 0.16 ± 0.06, 0.97 to 3.15 and 2.10 ± 0.70, 0.43 to 1.81 and 1.19 ± 0.46, 1.38 to 9.23 and 5.65 ± 2.53, 5.09 to 24.15 and 14.54 ± 6.12, 0.31 to 1.21 and 0.69 ± 0.30, 0.08 to 0.38 and 0.18 ± 0.11, 0.32 to 1.92 and 1.15 ± 0.58, 5.69 to 16.24 and 10.57 ± 3.58 for As, Cu, Cr, Cy, Fe, Mn, Ni, Pb, and Zn respectively. The observed heavy metal concentrations of the dumpsite are in the following trend: Fe $>$ Zn $>$ Cy $>$ Cu $>$ Cr $>$ Pb $>$ Mn $>$ Ni $>$ As. The heavy metal concentrations in comparison with established limits of the Federal Environmental Protection Agency, FEPA (1991), and the standard limit reported by Jolaosho (2024) are also presented in Table 5.

The arsenic concentration (0.16 ± 0.06) of the dumpsite’s leachate is greater than the limit of 0.05 prescribed by FEPA (1991). The Cu concentration is greater than the FEPA (1991) limit of 0.2 but within the $<$ 1 standard limit reported by Jolaosho (2024). The Cr concentration is greater than the FEPA (1991) limit of 0.05 but also within the limit of $<$ 1 standard limit reported by Jolaosho (2024). The Fe, Mn, Pb, and Zn concentration values were all above the respective FEPA (1991) limits of 5, 0.2, 0.1, and 2. However, Mn, Pb, and Zn were greater than the standard limits of 0.05, $<$ 1, and $<$ 1 reported by Jolaosho (2024) as the standard limits. The Fe concentration value fell within the maximum standard limit of 20. For Ni, the value was within the standard limit but met the requirement of FEPA (1991).

The coefficient of variation (CV) is obtained by dividing the standard deviation by the mean. It is used to compare the variability of the data, and when the CV is more than 10%, it indicates that the data values are far from the mean value. The obtained CV values are predominantly greater than 10% except for pH, Temperature, and COD. The variation in the concentration of heavy metals could be attributed to the difference in the grade of the selected sampling area, and other anthropogenic activities such as the age of the dumped waste, the decomposition rate of waste, and the type of dumped waste.

### Correlation between leachate physico-chemical and heavy metal parameters

The correlation that exists between a parameter and each paired parameter is presented in Table 6. Strong negative correlations were found between all parameters with pH, except BOD. Their values were $\ge$ —0.9. The correlation between BOD and pH has a positive correlation value of 0.90. Phenol, Ammonia, and Fecal Coliform have a nearly perfect negative correlation of −1.00, 0.99, and – 0.99, respectively. The extreme negative correlation (−1.00) that exists between pH and phenol indicates that phenol is pH-sensitive and is common in acidic environments. There are predominantly positive correlations between Temperature and other parameters, except for BOD, which had a negative moderate to strong correlation with other parameters. Temperature was found to have a strong positive correlation with Nitrate (0.90) and Ni (0.96). Also, the collinearity of AN, TKN, Ammonia, Phosphates, Phenol, and FCB is indicative that the leachate is a product of the dumping of MSW and probably sewage from industrial sources, leading to potential pollution.

**Table 6:** Correlation coefficient between the concentration of physico-chemical parameters and heavy metals of the dumpsite’s leachate

	*pH*	*Tem*	*TDS*	*T. Nit*	*TKN*	*AN*	*Phe*	*Chl*	*Sulp*	*Amm*	*Pho*	*Nit*	*BOD*
pH	1												
Temp	−0.90	1											
TDS	−0.88	0.60	1										
T. Nit	−0.96	0.79	0.85	1									
TKN	−0.97	0.81	0.87	1.00	1								
AN	−0.97	0.84	0.90	0.90	0.93	1							
Phen	−1.00	0.87	0.89	0.97	0.98	0.98	1						
Chlo	−0.93	0.69	0.99	0.91	0.93	0.95	0.95	1					
Sulph	−0.97	0.85	0.82	0.99	0.99	0.92	0.98	0.89	1				
Amm	−0.99	0.83	0.93	0.94	0.97	0.99	0.99	0.97	0.95	1			
Phos	−0.94	0.75	0.96	0.87	0.91	0.98	0.95	0.98	0.88	0.98	1		
Nit	−0.96	0.90	0.80	0.89	0.92	0.98	0.97	0.87	0.94	0.96	0.92	1	
BOD	0.90	−0.87	−0.80	−0.74	−0.79	−0.95	−0.89	−0.83	−0.78	−0.91	−0.92	−0.93	1
COD	−0.87	0.71	0.90	0.74	0.79	0.95	0.87	0.91	0.76	0.92	0.97	0.89	−0.96
T. Col	−0.91	0.72	0.98	0.83	0.86	0.94	0.91	0.97	0.82	0.95	0.98	0.85	−0.90
FCB	−0.99	0.88	0.88	0.93	0.95	0.99	0.99	0.93	0.95	0.99	0.96	0.99	−0.93
As	−0.88	0.69	0.94	0.77	0.81	0.95	0.88	0.94	0.78	0.93	0.98	0.87	−0.93
Cu	−0.95	0.76	0.95	0.90	0.93	0.99	0.97	0.98	0.91	0.99	0.99	0.94	−0.91
Cr	−0.84	0.63	0.93	0.73	0.77	0.93	0.85	0.92	0.73	0.91	0.97	0.85	−0.91
Cy	−0.87	0.67	0.93	0.79	0.83	0.96	0.89	0.94	0.81	0.94	0.98	0.89	−0.91
Fe	−0.93	0.78	0.92	0.84	0.88	0.99	0.94	0.95	0.86	0.97	0.99	0.94	−0.95
Mn	−0.95	0.88	0.86	0.82	0.86	0.97	0.94	0.89	0.85	0.95	0.96	0.95	−0.99
Ni	−0.94	0.96	0.74	0.81	0.84	0.93	0.92	0.80	0.86	0.91	0.88	0.96	−0.97
Pb	−0.93	0.75	0.96	0.86	0.88	0.91	0.92	0.96	0.84	0.94	0.95	0.83	−0.87
Zn	−0.98	0.86	0.93	0.91	0.93	0.98	0.98	0.96	0.91	0.99	0.98	0.94	−0.93

	*COD*	*T. Coli*	*FCB*	*As*	*Cu*	*Cr*	*Cy*	*Fe*	*Mn*	*Ni*	*Pb*	*Zn*	
pH													
Temp													
TDS													
T. Nit													
TKN													
AN													
Phen													
Chlo													
Sulph													
Amm													
Phos													
Nit													
BOD													
COD	1												
T. Col	0.95	1											
FCB	0.91	0.91	1										
As	0.99	0.97	0.91	1									
Cu	0.95	0.95	0.98	0.96	1								
Cr	0.99	0.95	0.88	1.00	0.95	1							
Cy	0.99	0.94	0.93	0.99	0.98	0.99	1						
Fe	0.99	0.96	0.97	0.99	0.99	0.97	0.99	1					
Mn	0.95	0.94	0.96	0.95	0.94	0.92	0.92	0.97	1				
Ni	0.87	0.84	0.95	0.85	0.88	0.81	0.83	0.91	0.97	1			
Pb	0.90	0.99	0.90	0.93	0.93	0.90	0.89	0.93	0.93	0.84	1		
Zn	0.93	0.97	0.97	0.94	0.97	0.91	0.92	0.97	0.98	0.93	0.97	1	

TDS is Total Dissolved Solids, T. Nit is Total Nitrogen, TKN is Total Kjeldahl Nitrogen, AN is Ammonia Nitrogen, Phen. Is Phenol, Chlo. is Chloride, Sulph. is Sulphate, Amm. is Ammonia, Phos. Phosphate, Nit. is Nitrate, BOD is Biological Oxygen Demand, COD is Chemical Oxygen Demand, T. Col. is Total Coliform, FCB is Faecal Coliform Bacteria, As is Arsenic, Cu is Copper, Cr is Chromium, Cy is Cyanide, Fe is iron, Mn is Manganese, Ni is Nickel, Pb is Lead, Zn is zinc

The inter-heavy metals correlation showed that they exhibit strong mutual correlations since they are all $\ge$ 0.9. The positive linear relationship exhibited by the paired heavy metals showed that they have a common source (Ojuri et al., 2018a, b). The microbiological parameters (Total coliform and Fecal Coliform bacteria) show strong correlations with nutrient parameters such as TKN, Ammonia nitrogen, and Phosphates, as well as organic parameters (BOD and COD), which are also indicative of domestic or fecal contamination.

The fact that the heavy metal, nutrient, organic matter, and acidity parameters are interlinked (correlated) suggests that the dumpsite receives solid and sewage from domestic, agricultural, and industrial sources. Generally, many parameters are strongly interrelated ($\ge$ 0.9), suggesting that the sources contributing to the parameters' occurrence and load are common and interconnected*.*

### Chemical and heavy metal concentration of the dumpsite soil

The chemical and heavy metal concentrations in Igbatoro dumpsite soil are represented in Tables 7 and 8, respectively. The table shows the distribution of both the chemical and heavy metals in the dumpsite. The concentration of the chemical parameters ranges from 8.25 to 45.8, 24.75 to 85.55, 28.35 to 87.35, and 3.95 to 17.78 for Na, Ca, K, and Mg respectively while the concentration of heavy metals in the dumpsite ranges from 0.09 to 1.75, 0.21 to 2.17, 0.06 to 0.95, 128.59 to 1058.26, 0.26 to 1.74, 0.07 to 0.72, 0.19 to 1.66, and 8.75 to 23.81 for Cd, Cr, Co, Fe, Mn, Ni, Zn and Pb respectively. The comparison of the mean concentration of the chemical parameters with the control site samples (reference values) shows that the dumping of waste on the dumpsite must have contaminated the soil. The mean concentrations of Na of the dumpsite soil are 15.14, 18.50, and 21.34 for the dumpsite underlying soil, while the control site values are 8.35, 9.55, and 10.55 for depths 0.5 m, 1.0 m, and 1.5 m, respectively. The Na concentrations at 0.5, 1.0, and 1.5 m depths are 1.8, 1.9, and 2.0 times the control samples. For Ca, the mean concentration at 0.5, 1.0, and 1.5 m depths are also 2.0, 1.7, and 1.5 times the control samples. For K, their mean concentrations at 0.5, 1.0, and 1.5 m depths are 1.9, 1.8, and 1.9 times the control samples, respectively. The Mg mean concentration at 0.5, 1.0, and 1.5 m depths are 1.8, 1.6, and 1.5 times the control samples, respectively.

**Table 7:** Chemical parameters of Igbatoro dumpsite soil and the control site

Location	Na	Ca	K	Mg
1A	11.50 ± 0.30	43.15 ± 0.15	52.8 ± 0.10	4.774 ± 0.004
1B	13.4 ± 0.10	50.45 ± 0.25	58.60 ± 0.30	6.189 ± 0.004
1C	15.80 ± 0.10	62.10 ± 0.20	75.35 ± 0.25	3.951 ± 0.004
2A	10.35 ± 0.05	24.75 ± 0.05	28.35 ± 0.15	9.624 ± 0.006
2B	18.10 ± 0.10	37.65 ± 0.25	33.65 ± 0.05	9.247 ± 0.003
2C	19.70 ± 0.20	40.45 ± 0.25	35.15 ± 0.15	9.817 ± 0.001
3A	14.40 ± 0.10	49.05 ± 0.05	69.50 ± 0.30	12.314 ± 0.003
3B	13.40 ± 0.20	52.35 ± 0.15	66.60 ± 0.20	13.197 ± 0.002
3C	15.20 ± 0.20	54.55 ± 0.05	70.70 ± 0.10	12.878 ± 0.002
4A	17.55 ± 0.05	35.75 ± 0.15	46.30 ± 0.30	6.154 ± 0.006
4B	20.45 ± 0.25	47.35 ± 0.15	45.25 ± 0.15	8.62 ± 0.004
4C	21.55 ± 0.05	53.15 ± 0.15	52.75 ± 0.05	8.95 ± 0.003
5A	8.25 ± 0.15	32.50 ± 0.00	37.55 ± 0.35	6.514 ± 0.004
5B	10.55 ± 0.25	38.55 ± 0.15	42.75 ± 0.15	7.262 ± 0.004
5C	11.80 ± 0.10	39.85 ± 0.05	50.20 ± 0.20	9.61 ± 0.003
6A	29.40 ± 0.10	82.15 ± 0.15	62.55 ± 0.05	13.291 ± 0.003
6B	36.40 ± 0.20	72.65 ± 0.25	86.50 ± 0.20	14.783 ± 0.003
6C	45.80 ± 0.10	85.55 ± 0.05	87.35 ± 0.15	17.782 ± 0.002
7A	14.50 ± 0.30	52.20 ± 0.10	61.75 ± 0.05	10.788 ± 0.002
7B	17.19 ± 0.00	60.50 ± 0.30	60.50 ± 0.20	12.767 ± 0.013
7C	19.55 ± 0.15	56.55 ± 0.05	64.40 ± 0.20	9.789 ± 0.015
CA	8.35 ± 0.15	22.60 ± 0.30	26.65 ± 0.15	5.154 ± 0.006
CB	9.55 ± 0.05	30.35 ± 0.15	31.15 ± 0.15	6.62 ± 0.004
CC	10.55 ± 0.25	37.55 ± 0.25	32.55 ± 0.15	6.865 ± 0.004

**Table 8:** Heavy metal concentrations of Igbatoro dumpsite and the control site in mg/kg

Location	Cd	Cr	Co	Cu	Fe	Mn	Ni	Pb	Zn
1A	0.116 ± 0.002	0.324 ± 0.004	0.081 ± 0.003	0.431 ± 0.002	197.22 ± 0.01	0.596 ± 0.003	0.087 ± 0.001	0.432 ± 0.004	10.612 ± 0.002
1B	0.136 ± 0.001	0.387 ± 0.002	0.093 ± 0.003	1.059 ± 0.002	218.583 ± 0.007	0.712 ± 0.002	0.092 ± 0.002	0.475 ± 0.005	13.512 ± 0.004
1C	0.147 ± 0.002	0.413 ± 0.003	0.118 ± 0.001	1.211 ± 0.003	292.757 ± 0.003	0.761 ± 0.002	0.117 ± 0.002	0.519 ± 0.003	14.249 ± 0.002
2A	0.256 ± 0.002	0.617 ± 0.003	0.156 ± 0.002	0.723 ± 0.001	278.146 ± 0.004	0.376 ± 0.002	0.074 ± 0.004	0.628 ± 0.002	18.207 ± 0.002
2B	0.198 ± 0.002	0.642 ± 0.003	0.134 ± 0.002	1.396 ± 0.002	253.745 ± 0.025	0.632 ± 0.002	0.095 ± 0.001	0.747 ± 0.002	17.584 ± 0.006
2C	0.275 ± 0.004	0.721 ± 0.009	0.209 ± 0.003	1.820 ± 0.006	381.283 ± 0.007	0.681 ± 0.004	0.137 ± 0.004	0.785 ± 0.003	18.222 ± 0.008
3A	0.261 ± 0.003	0.531 ± 0.003	0.183 ± 0.003	0.816 ± 0.002	404.3 ± 0.004	0.416 ± 0.003	0.163 ± 0.003	0.414 ± 0.003	16.273 ± 0.053
3B	0.295 ± 0.001	0.576 ± 0.002	0.210 ± 0.001	1.516 ± 0.003	460.783 ± 0.001	0.563 ± 0.002	0.216 ± 0.001	0.607 ± 0.003	15.845 ± 0.003
3C	0.373 ± 0.003	0.839 ± 0.002	0.311 ± 0.003	1.684 ± 0.004	504.271 ± 0.003	0.709 ± 0.003	0.347 ± 0.003	0.674 ± 0.003	16.909 ± 0.003
4A	0.091 ± 0.004	0.207 ± 0.003	0.056 ± 0.004	0.216 ± 0.002	128.592 ± 0.011	0.258 ± 0.001	0.105 ± 0.003	0.252 ± 0.009	9.778 ± 0.003
4B	0.167 ± 0.003	0.322 ± 0.003	0.094 ± 0.002	0.533 ± 0.003	158.293 ± 0.002	0.393 ± 0.005	0.215 ± 0.004	0.286 ± 0.002	11.35 ± 0.002
4C	0.194 ± 0.004	0.368 ± 0.002	0.146 ± 0.002	0.606 ± 0.004	176.299 ± 0.006	0.517 ± 0.002	0.177 ± 0.002	0.44 ± 0.003	12.073 ± 0.007
5A	0.183 ± 0.002	0.351 ± 0.001	0.136 ± 0.003	0.217 ± 0.002	258.71 ± 0.002	0.686 ± 0.004	0.163 ± 0.002	0.389 ± 0.004	14.168 ± 0.003
5B	0.21 ± 0.002	0.376 ± 0.003	0.166 ± 0.002	0.393 ± 0.001	306.254 ± 0.006	0.85 ± 0.004	0.186 ± 0.002	0.415 ± 0.003	15.277 ± 0.003
5C	0.334 ± 0.006	0.394 ± 0.001	0.188 ± 0.003	0.483 ± 0.003	514.518 ± 0.002	0.874 ± 0.004	0.273 ± 0.003	0.487 ± 0.004	14.974 ± 0.002
6A	1.282 ± 0.003	1.039 ± 0.003	0.421 ± 0.003	1.812 ± 0.002	917.216 ± 0.002	1.287 ± 0.004	0.52 ± 0.003	1.021 ± 0.003	19.208 ± 0.004
6B	1.606 ± 0.004	1.282 ± 0.003	0.614 ± 0.004	2.091 ± 0.003	975.742 ± 0.008	1.609 ± 0.004	0.586 ± 0.002	1.371 ± 0.002	22.511 ± 0.002
6C	1.752 ± 0.001	2.168 ± 0.002	0.95 ± 0.002	2.626 ± 0.004	1058 ± 0.011	1.741 ± 0.003	0.717 ± 0.001	1.655 ± 0.005	23.807 ± 0.003
7A	0.289 ± 0.003	0.425 ± 0.003	0.314 ± 0.003	0.166 ± 0.005	172.282 ± 0.004	0.37 ± 0.002	0.115 ± 0.002	0.195 ± 0.002	8.748 ± 0.004
7B	0.448 ± 0.002	0.459 ± 0.002	0.533 ± 0.007	0.204 ± 0.003	226.568 ± 0.017	0.383 ± 0.003	0.148 ± 0.003	0.217 ± 0.002	9.515 ± 0.005
7C	0.494 ± 0.002	0.513 ± 0.003	0.595 ± 0.001	0.246 ± 0.004	269.132 ± 0.004	0.394 ± 0.002	0.164 ± 0.002	0.254 ± 0.004	9.82 ± 0.003
CA	0.078 ± 0.002	0.094 ± 0.001	0.023 ± 0.023	0.107 ± 0.003	108.224 ± 0.006	0.221 ± 0.003	0.09 ± 0.004	0.134 ± 0.004	8.367 ± 0.002
CB	0.163 ± 0.003	0.137 ± 0.003	0.087 ± 0.003	0.128 ± 0.002	136.61 ± 0.004	0.304 ± 0.002	0.113 ± 0.003	0.159 ± 0.003	6.958 ± 0.012
CC	0.186 ± 0.002	0.153 ± 0.003	0.117 ± 0.001	0.148 ± 0.001	182.715 ± 0.004	0.395 ± 0.004	0.131 ± 0.001	0.194 ± 0.004	12.185 ± 0.005
Austria*	5	100	-	100	-		100	100	300
Canada*	8	75		100	-		100	200	400
Japan*	-	-	-	125	-	-	100	400	250
Great Britain*	3	50	-	100	-	-	50	100	300
Germany*	2	200	-	50	-	-	100	500	300
Nigerian**	0.8	100	-	36	-	437	35	85	140
WHO/FAO***	0.8–3	-	50	30 −36	100–1000	200	35	50—85	50—300

A is for 0.5 m, B is for 1.0 m, C is for 1.5 m depth of sample collection, * is Olanipekun et al. (2024), ** is DPR (2002 and 2018) and *** is (Gyabaah et al., 2023; Kolawole et al., 2023)

The concentration for the control samples at different depths ranges from 8.35 to 10.55, 22.60 to 37.55, 26.65 to 32.55, 5.15 to 6.86, 0.08 to 0.19, 0.09 to 0.15, 0.02 to 0.12, 108.22 to 182.71, 0.22 to 0.39, 0.09 to 0.13, 0.13 to 0.19, and 6.96 to 12.19 for Na, Ca, K, Mg, Cd, Cr, Co, Fe, Mn, Ni, Zn, Pb respectively. The heavy metal levels for the control samples are lower than the dumpsite samples due to the activities of the waste on the dumpsite. Predominantly, the deeper the depth, the higher the elemental concentration. The abundance of the heavy metals investigated within the dumpsite shows that Fe $>$ Zn $>$ Mn $>$ Cr $>$ Pb $>$ Cd $>$ Co $>$ Ni, while the abundance of the heavy metals investigated for the control site samples shows that Fe $>$ Zn $>$ Mn $>$ Pb $>$ Cd $>$ Cr $>$ Ni $>$ Co. Location 6 (6A, 6B, and 6 C) have the highest concentration of all elements. It indicates the location is heavily contaminated. However, the values of the heavy metals across the locations and various depths are predominantly lower than the permissible limits of the national and international standards presented in Table 8. There are exceptions for Cd at 6 A, 6B, and 6 C, while at 6 C, the Fe concentration was higher than the permissible limits.

The highest abundance of Fe and Zn is consistent with the previous findings (Aralu et al., 2024; El Fadili et al., 2022; Hussein et al., 2021; Tovide et al., 2025). The abundance of the five (5) top heavy metals found on the dumpsite is consistent with the heavy metal concentrations of related studies presented in Table 9. The abundance of heavy metals indicated the following trend: Fe > > Mn > Zn > Pb ≈ Cr > Cu ≈ Ni > Cd > Co.

**Table 9:** Heavy metal concentrations of some selected dumpsite soils in mg/kg

Cu	Pb	Cd	Mn	Ni	Zn	Cr	Fe	Co	Remark	References
10.85	10.38	0.91	273.76	3.53	62.04	0.72	ND	ND	0.3 cm	(Oguntuase et al., 2024)
1.63	2.07	2.21	10.8	2.69	8.34	2.69	9.89	1.41	Wet season (0.3 cm)	(Aralu et al., 2025)
2.65	3.18	4.42	11.8	4.84	8.62	2.89	12.4	2.38	Dry Season (0.3 cm)	
2.75	3.37	2.66	7.41	3.57	10.6	3.32	13.1	2.17	Wet season (0.3 cm)	(Aralu et al., 2024)
3.56	4.06	3.4	7.62	3.71	7.03	3.91	15.1	3.34	Dry season (0.3 cm)	
13.32	22.01	1.77	ND	8.50	42.51	21.16	406.27	ND	5–20 cm	(El Fadili et al., 2022)
12.2	2.8	4.41	ND	ND	222	ND	125	ND	UD (0 cm)	(Gyabaah et al., 2023)
35.6	5.24	3.65	ND	ND	356	ND	225	ND	0.5 UD (0.5 cm)	
21.6	4.9	2.34	ND	ND	339	ND	1150	ND	1.5 UD (1.5 cm)	
1.6	6.9	0.62	ND	ND	12.9	ND	1179	ND	0 PUD (0 cm)	
1.4	7.0	0.50	ND	ND	11.4	ND	1360	ND	0.5 PUD (0.5 cm)	
1.8	6.3	0.35	ND	ND	4.6	ND	317	ND	1.5 PUD (1.5 cm)	
48.05	23.02	1.36	92.82	15.34	65.21	346.82	58123	ND	D1 (10–50 cm)	(Hussein et al., 2021)
7.63	10.91	0.47	29.35	14.50	71.30	95.63	36158	ND	D2 (10–50 cm)	
61.63	46.79	-	176.97	12.16	61.25	101.84	55733	ND	D3 (10–50 cm)	
4.43	171.72	1.33	693.13	1.17	96.70	8.38	20190	ND	D4 (10–50 cm)	
0.40	17.77	1.88	178.82	1.98	86.86	12.36	26329	ND	D5 (10–50 cm)	

ND is not determined, UD is Urban Dumpsite; PUD is Peri-Urban Dumpsite

The mean of all the heavy metal concentrations in comparison with the reference value showed a considerable level of contamination. The mean concentrations of Cd at 0.5, 1.0, and 1.5 m depths were 4.4, 2.8, and 2.7 times higher than the control site. For Cr, the mean concentration at depths of 0.5, 1.0, and 1.5 m was 5.6, 4.1, and 5.1 times higher than the control sites. The mean concentration of Co at depths of 0.5, 1.0, and 1.5 m was 9.5, 2.9, and 3 times higher than the control sites. Similar were the observations with Fe and Mn at depths 0.5, 1.9, and 1.5 m, respectively. At 0.5 m depth, the mean concentrations of Fe and Mn were 3.1 and 2.6 times higher than the control site, respectively. Also, the concentrations of Fe and Mn were 2.7 and 2.4 times higher than the control site for 1.0 m depth, while they were 2.5 and 2.1 times higher than the control site for 1.5 m depth. The mean concentrations of Mn were on average 2 times higher than the control sites for depths 0.5, 1.0, and 1.5 m. The concentration of Pb at depths 0.5, 1.0, and 1.5 m were on average 3.6 times higher than the control site. For Zn, it was observed that the concentrations were 1.7, 2.2, and 1.3 times higher than the control site for depths of 0.5, 1.0, and 1.5 m, respectively. The concentration of the other chemical parameters and heavy metals of the dumpsite soil is higher than that of the control site, consistent with the findings of El Fadili et al. (2022). The highest concentration of Fe may be attributed to the fact that natural soil contains considerable iron, and not solely due to the dumping of waste on the dumpsite (Ihedioha et al., 2017). The loads of heavy metal concentration obtained from the dumpsite’s soil area result of the dumping of municipal waste on the dumpsite (Agbeshie et al., 2020; Okeke et al., 2024).

The statistical analysis presented in Table 10 showed the relationship (inter-relationship) between the elements studied on the dumpsite and the dumpsite’s depth, as well as the location. The p-values obtained between the elements and the location are predominantly lower than 0.05. Also, the F values are all greater than Fcrit for the inter-relationship between the parameters and location. Except for the inter-relationship between Mg and depth that has an F value less than Fcrit (3.1934) and also a P-value greater than 0.05, all other F values are greater than the Fcrit, and P-values are less than 0.05. These statistical outputs indicate that there is a statistical significance between the parameters and location, as well as the parameters and depth.

**Table 10:** ANOVA summary of the effect of location and depth on the chemical parameters of the soil

Parameter	Location			Depth		
	F	Fcrit	p-value	F	Fcrit	*p*-value
Na	33.6316	2.7642	0.000000105	9.5155	3.7389	0.0025
Ca	33.6316	2.7642	0.000000105	9.5155	3.7389	0.0025
K	31.1176	2.7642	0.000000176	7.2678	3.7389	0.0068
Mg	24.5469	2.7642	0.000000786	3.1934	3.7389	0.072
Cd	109.9239	2.7642	0.0000000000378	7.1455	3.7389	0.0073
Cr	13.6812	2.7642	0.0000276	3.3103	3.7389	0.0665
Co	16.8113	2.7642	0.00000814	6.4968	3.7389	0.0101
Fe	158.7505	2.7642	0.00000000000304	19.4455	3.7389	0.000091
Mn	100.0287	2.7642	0.0000000000721	22.8277	3.7389	0.0000392
Ni	58.3303	2.7642	0.00000000277	11.9217	3.7389	0.001
Pb	46.6132	2.7642	0.0000000191	7.7619	3.7389	0.0054
Zn	41.0339	2.7642	0.0000000286	6.2014	3.7389	0.0118

### Assessment of the dumpsite’s leachate

#### Leachate contamination potential of igbatoro dumpsite

**Fig. 2:**
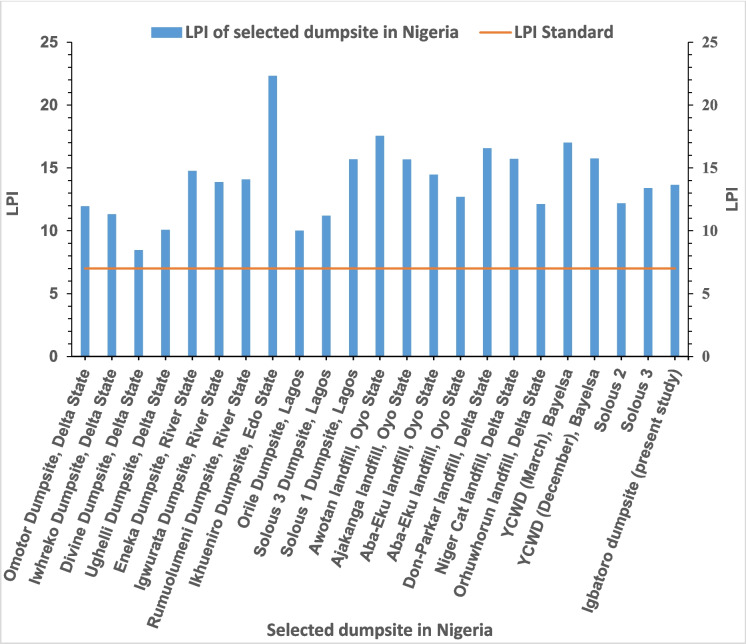
Comparison of pollution indices of landfills from various locations in Nigeria

The contamination potential of the Igbatoro dumpsite using the LPI tool is presented in Table 11. The sub-LPI of the organic parameter of the leachate is 11.559, while the inorganic and heavy metal parameters have values of 5.206 and 18.853, respectively. The obtained overall LPI of the Igbatoro dumpsite is 13.65. The obtained value is greater than the threshold (LPI standard value of 7.378) often used, especially by some developing countries (Rajoo et al., 2020), and also greater than the LPI of 10, indicating a hazardous nature of the dumpsite and potential contamination of surrounding groundwater have also been promoted (Afolabi et al., 2022; Jolaosho, 2024). This indicates that the leachate from the dumpsite has contamination potential. This is attributable to the lack of clay liner and leachate collection systems, which are typical of unsanitary landfills (open dumpsites), allowing for migration of the leachate through the soil, and leaching to the groundwater or nearby surface water. Studies conducted in Nigeria, presented in Fig. 2, revealed that the groundwater and soil in the vicinity of disposal sites may have been contaminated. The LPI of the present study is within the range of values for dumpsites, especially in Nigeria.

**Table 11:** Computed overall LPI values for the Igbatoro landfill

Parameters	Conc	wi (Ov.)	wi	pi	Ov. LPI (wipi)	LPI(wipi)
COD	16.58	0.267	0.062	5.1	1.3617	0.316
BOD	8.88	0.263	0.061	3	0.789	0.183
Phenolic Compounds	0.48	0.246	0.057	0	0	0.000
Total coliform bacteria	61	0.224	0.052	42	9.408	2.184
LPI_or_					*11.5587*	
pH	6.03	0.214	0.055	5	1.07	0.275
TKN	70.3	0.206	0.053	6	1.236	0.318
Ammonia Nitrogen	10.29	0.198	0.051	5	0.99	0.255
TDS	369.99	0.195	0.050	5	0.975	0.250
Chloride	49.17	0.187	0.048	5	0.935	0.240
LPI_in_					*5.206*	
Total Chromium	1.26	0.125	0.064	7	0.995449	0.448
Lead	1.19	0.123	0.063	10	1.399317	0.630
Mercury	ND	0.121	0.062	ND	-	-
Arsenic	0.17	0.119	0.061	5	0.676906	0.305
Cyanide	5.82	0.114	0.058	98	12.7099	5.684
Zinc	10.65	0.11	0.056	7	0.875995	0.392
Nickel	0.18	0.102	0.052	5	0.580205	0.260
Copper	2.09	0.098	0.050	10	1.114903	0.500
Iron	14.91	0.088	0.045	5	0.500569	0.225
LPI_hm_					*18.85324*	
**LPI**						**13.289**
**Overall LPI**						**13.654**

Revised leachate pollution index (R-LPI) of Igbatoro dumpsite.

Eleven parameters make up the r-LPI, while there are nine (9) parameters common to the LPI and the r-LPI. Herbicides and FCBs were added to the r-LPI in addition to the nine common parameters. The R-LPI and the computation of Igbatoro dumpsite parameters are shown in Table 12. R-LPI helps determine the leachate's polluting potential only based on the concentration of contaminants present, not the volume. Three groups, including toxicants, basic pollutants, and heavy metals, were used as the criteria for the r-LPI computation. Studies on the use of r-LPI are still limited.

**Table 12:** Computation of revised Leachate Pollution Index (r-LPI) of Igbatoro dumpsite

r-LPI class	Parameters	Concentration	wi	pi	wipi
r-LPI _Tox_	Cyanide	5.82	0.451	100	45.1
	Pesticides	ND	0.299	0	0
	Phenolic C	0.48	0.251	6	1.506
					**66.390**
r-LPI _Hm_	Mercury	ND	0.374	0	0
	Lead	1.19	0.255	41	10.455
	Arsenic	0.17	0.231	8	1.848
	Total chromium	1.26	0.14	9	1.26
				58	**21.666**
r-LPI _Bp_	FCB	22	0.305	75	22.875
	COD	16.58	0.278	7	1.946
	BOD	8.88	0.24	4	0.96
	pH	6.03	0.176	15	2.64
				101	**28.421**
Overall r-LPI					**40.397**

The r-LPI value of 40.39 for the Igbatoro dumpsite leachate indicated pollution potential. The value obtained for r-LPI was higher than the LPI, similar to the findings of Wdowczyk et al. (2024). The findings of Bisht et al. (2022), the pioneering research team, showed that the LPIs of Bhalswa, Okhla, and Ghazipur, which were 29.295, 25.874, and 27.728, respectively, increased to 38.969, 44.427, and 46.288 when the r-LPI was used. This showed that the inclusion of Herbicides and FCBs into the pollution indices contributed to the leachate contamination load. Also, the increase in the value of the r-LPI may be attributed to the increased pollutant weight factors ascribed to the leachate parameters.

### Pollution indices of Igbatoro dumpsite’s soil

#### Geo accumulation index (Igeo) of Igbatoro dumpsite

**Fig. 3:**
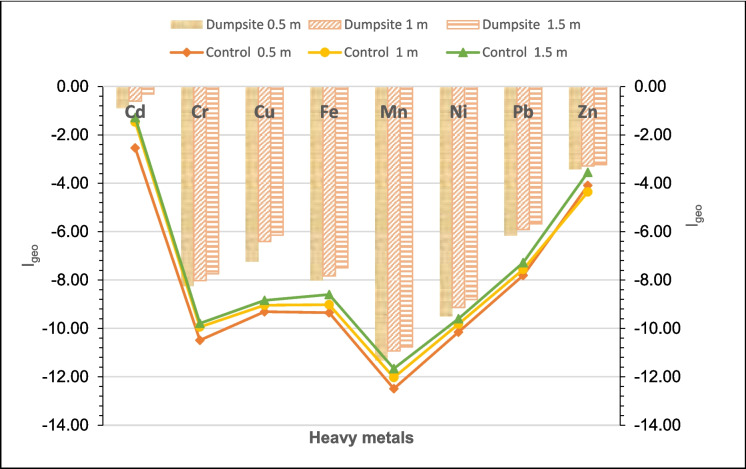
The mean I_geo_ values of the sampled Igbatoro dumpsite soil heavy metals at varying depths

Results obtained for the geo-accumulation index (Igeo) of the heavy metals in the dumpsite soil are presented in Table S1. The Igeo values are −0.60 for Cd, −8.00 for Cr, −6.60 for Cu, −7.78 for Fe, −11.00 for Mn, −9.15 for Ni, −5.92 for Pb, and −3.314 for Zn. Although the obtained values are all in class 0, i.e., they are not polluted by the heavy metals(Uncontaminated), their comparison with control site soil samples indicated a considerable pollution level higher than the control samples. The I_geo_values of all the heavy metals are greater than the control site soil samples. The I_geo_ values of the heavy metals decrease in the following order: Cd (−0.60) $>$ Zn (−3.31) $>$ Pb (−5.92) $>$ Cu (−6.60) $>$ Fe (−7.78) $>$ Cr (−8.00) $>$ Ni (−9.15) $>$ Mn (−11.00). The highest I_geo_ obtained from Cd is consistent with the study of El Fadili et al. (2022). The variation in the I_geo_ of the heavy metals in relation to the depth (Fig. 3) also showed that the deeper the depth, the higher the I_geo_ for all the heavy metals. For depth 0.5 m, the I_geo_ values of the heavy metals decrease in the following order: Cd (−0.90) $>$ Zn (−3.42) $>$ Pb (−6.17) $>$ Cu (−7.24) $>$ Fe (−8.01) $>$ Cr (−8.25) $>$ Ni (−9.51) $>$ Mn (−11.31). The decreasing trend of I_geo_ values of the heavy metals is in a similar order of Cd $>$ Zn $>$ Pb $>$ Cu $>$ Fe $>$ Cr $>$ Ni $>$ Mn, both for depths 1.0 and 1.5 m.

The obtained highest I_geo_ for cadmium (Cd) is similar to the previous findings (El Fadili et al., 2022; Ihedioha et al., 2017; Olanipekun et al., 2024). Also, the studies of Ogundele et al. (2019) and Ogundele et al. (2020) had the I_geo_ values of Cd to be greater than those of other heavy metals in the studied dumpsites in Southwest Nigeria. This points to the fact that cadmium is a leading pollutant found in Nigerian dumpsites. The obtained I_geo_ values of the Igbatoro dumpsite differ from the findings of Olanipekun et al. (2024), who also studied the Igbatoro dumpsite among others. However, the disparity may be due to the difference in the depth of soil sample collection.

#### Metal pollution index (MPI), pollution load index, and degree of contamination of the Igbatoro dumpsite underlying soil

**Fig. 4:**
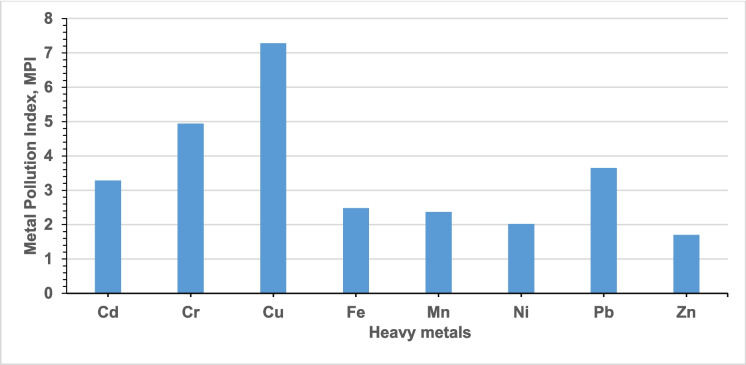
Metal pollution index of igbatoro dumpsite

**Fig. 5:**
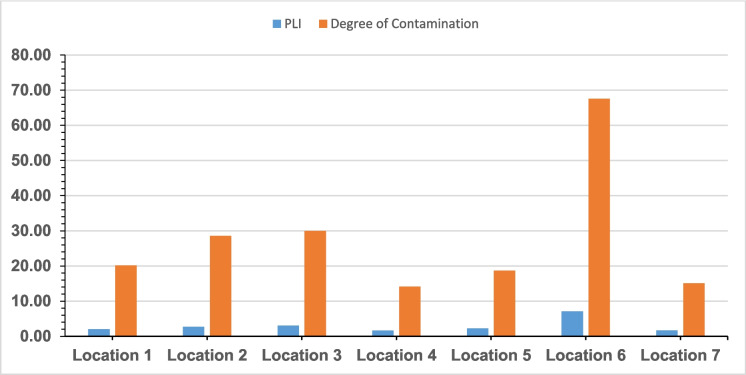
PLI and degree of contamination of Igbatoro dumpsite

The assessment of the overall contamination of the Igbatoro dumpsite based on the metal pollution index, pollution load index, and degree of contamination is shown in Table S2, Figs. 4 and 5. The MPI of the individual heavy metal across all the studied locations of the dumpsite showed that location 6 (P6 0.5 m, P6 1.0 m, and P6 1.5 m) has the highest. The MPI has the following decreasing trend: Cu (7.28) $>$ Cr (4.94) $>$ Pb (3.65) $>$ Cd (3.28) $>$ Fe (2.48) $>$ Mn (2.37) $>$ Ni (2.02) $>$ Zn (1.70). Comparing the values with the available limits, the contamination of the dumpsite soil with Cu and Cr is severe, while Pb, Cd, Fe, and Mn have moderately polluted the dumpsite soil. Ni and Zn have only slightly polluted the dumpsite soil. The mean MPI of all the studied heavy metals present in the dumpsite showed that they all fell within the pollution class ($\ge$ 1.1), implying that their presence poses a risk to the environment, plants, and soil. The pollution status of Fe, Mn, Ni, and Zn agrees with the findings of Oluwatuyi et al. (2020) for dumpsites in Akure, Southwest Nigeria. The PLI outlook of the dumpsite showed, as expectedly, that location 6 (P6 0,5 m, P6 1.0 m, and P6 1.5 m) has the highest, which invariably resulted in the highest degree of contamination. The PLI decreasing trend in terms of location and depth is as follows: P6 0.5 m (7.94) $>$ P6 1.0 m (7.33) $>$ P6 1.5 m (5.95) $>$ P3 0.5 m (3.21) $>$ P3 1.0 m (3.14) $>$ P2 0.5 m (2.93) $>$ P3 1.5 m (2.88) $>$ P2 1.0 m (2.65) $>$ P2 1.5 m (2.50) $>$ P5 0.5 m (2.43) $>$ P5 1.0 m (2.25) $>$ P1 0.5 m (2.12) $>$ P5 1.5 m (2.08) $>$ P1 1.0 m (2.08) $>$ P1 1.5 m (1.88) $>$ P7 0.5 m (1.85)$>$ P4 1.0 m (1.74) $>$ P7 1.0 m (1.72) $>$ P4 1.5 m (1.69) $>$ P7 1.5 m (1.54) $>$ P4 0.5 m (1.44). The PLI outlook also showed that all the PLI values reduced with the depth of soil sample collection, except for point 4. Also, the degree of contamination of the dumpsite indicated that the $\sum MPI$ followed the decreasing trend based on location:P6 0.5 m $>$ P6 1.0 m $>$ P6 1.5 m $>$ P3 1.0 m $>$ P3 1.5 m $>$ P3 0.5 m $>$ P2 0.5 m $>$ P2 1.0 m $>$ P2 1.5 m $>$ P1 1.0 m $>$ P5 0.5 m $>$ P1 1.5 m $>$ P1 0.5 m $>$ P5 1.0 m $>$ P5 1.5 m $>$ P7 0.5 m $>$ P4 1.0 m $>$ P4 1.5 m $>$ P7 1.0 m $>$ P7 1.5 m $>$ P4 0.5 m.

Points 1, 2, 3, 5, and 7 predominantly indicated a significant degree of contamination, while Point 4 exhibited a slight degree of contamination. Point 6 showed a high degree of contamination. Overall, the evaluation of the concentration of the studied heavy metals gives it away as having a significant degree of contamination since the mean MPI of the dumpsite is within 16 $<$ C_deg_ $<$ 32.

#### Ecological risk factor (E^i^r) and potential ecological risk factor (RI) of Igbatoro dumpsite

The ecological and potential ecological risk factor of the Igbatoro dumpsite is presented based on the metal pollution index, pollution load index, and degree of contamination, and are shown in Table S3. The E^i^r of the individual heavy metal across all the studied locations of the dumpsite showed that location 6 (P6 0.5 m, P6 1.0 m, and P6 1.5 m) has the highest. Generally, the E^i^r of Cd ranges from 23.13 to 480.56 and has the highest mean of 98.34, while Cr ranges from 4.59 to 28.90 and has a mean of 9.88. The Cadmium highest E^i^r is similar to the findings of Olanipekun et al. (2024). The ranges of Cu, Ni, Pb, and Zn are 7.52 to 87.53, 4.08 to 28.89, 6.68 to 43.55, and 0.81 to 3.23, respectively. The cadmium level of the dumpsite soil has an appreciable ecological risk. Cadmium is a toxicant that affects living cells and poses a high risk to humans (Ebong et al., 2019). The ecological risk (E^i^r) of the other heavy metals showed that they are of low ecological risk. However, the levels of these E^i^r may put them in the low ecological risk class; their concentrations may be a risk concern as humans will be exposed to the soil either directly or indirectly. The E^i^r of Igbatoro dumpsite has the following decreasing trend: Cd (98.34) $>$ Cu (36.42) $>$ Pb (18.25)$>$ Ni (10.10) $>$ Cr (9.88) $>$ Zn (1.70).

**Fig. 6:**
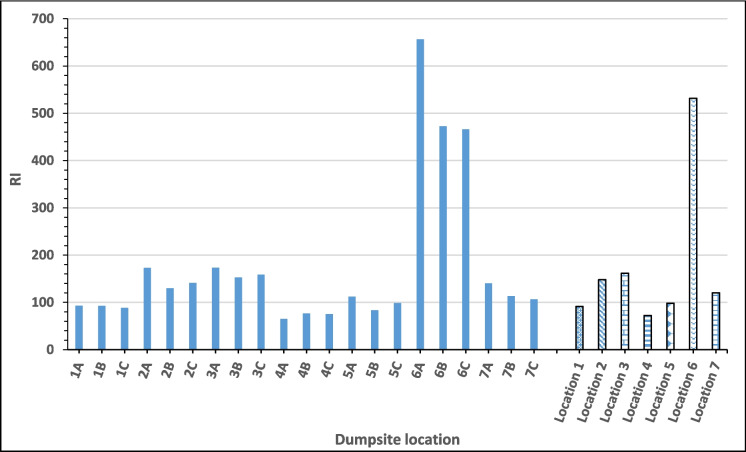
The potential ecological risk of different locations of Igbatoro dumpsite

The RI of all the locations (Fig. 6) followed the decreasing trend: P6 0.5 m (656.47) $>$ P6 1.0 m (472.55) $>$ P6 1.5 m (466.07) $>$ P3 0.5 m (173.44) $>$ P2 0.5 m (172.79) $>$ P3 1.5 m (158.63) $>$ P3 1.0 m (152.80) $>$ P2 1.5 m (141.02) $>$ P7 0.5 m (140.26) $>$ P2 1.0 m (130.04) $>$ P7 1.0 m (113.23) $>$ P5 0.5 m (111.96) $>$ P7 1.5 m (106.82) $>$ P5 1.5 m (98.60) $>$ P1 0.5 m (92.80) $>$ P1 1.0 m (92.60) $>$ P1 1.5 m (88.33) $>$ P5 1.0 m (83.37) $>$ P4 1.0 m (76.61) $>$ P4 1.5 m (75.09) $>$ P4 0.5 m (65.03). Comparatively, the RI of the six locations (1, 2, 3, 4, 5, and 6) also indicated the following decreasing trend: Location 6 (531.70) $>$ Location 3 (161.62) $>$ Location 2 (147.95) $>$ Location 5 (97.98) $>$ Location 1 (91.30) $>$ Location 4 (72.24). Locations 3 and 6 have their RI greater than 150 but less than 300, indicating that the heavy metals contamination at these locations poses moderate ecological risk, while others are of low ecological risk since they are less than 150. Further analysis also showed that the RI in terms of depth is inconsistent with a depth of 0.5 m across the locations, having a mean RI of 201.85, and a depth of 1.0 m has a mean RI of 160.17, while a depth of 1.5 m has a mean RI of 162.08. This implies that the surface depth (0.5 m) poses the highest potential ecological risk (RI), although all the locations’ potential ecological risk (RI) fell within the moderate ecological risk class.

The potential ecological risk (RI) of the dumpsite of 174.70 is greater than the RI reported by Essien et al. (2019) for a Nigerian dumpsite soil evaluated during the wet (rainy season). It is, however, less than the 276.61 obtained during the dry season. The RI of the Igbatoro dumpsite is also lower than the RI in the findings of Ogundele et al. (2020), who also reported a RI of 285.5 for a dumpsite in Ondo City, Nigeria. The RI of these dumpsites, however, categorizes them (including the present study) as having a moderate ecological risk.

## Conclusion

The leachate characteristics of the Igbatoro dumpsite, through the assessment of twenty-five parameters, indicated that varying concentrations of the parameters exceeded permissible limits, demonstrating environmental risk and public health concerns. The correlations among various parameters of the leachate confirm that the distribution of parameters is significantly influenced by one another. The analysis of dumpsite soil samples also reveals significant contamination from both chemical parameters and heavy metals compared to control samples. The statistical analysis of the dumpsite soil indicates a predominantly significant relationship between the studied elements at the dumpsite and both depth and location. An overall LPI of 13.65 and r-LPI of 40.39 indicate potential risks of contamination for the surrounding surface and groundwater. The pollution indices of the dumpsite through assessment of the I_geo_ showed that the overall pollution levels are of an uncontaminated category (class 0), although higher than the control site. The MPI presented significant contamination levels, particularly at location 6. The PLI further confirms the high contamination levels at location 6, with a notable decrease in PLI values with soil depth, except at point 4. The degree of contamination of the dumpsite ranges from considerable to a high degree of contamination, with the location exhibiting a high degree.

The ecological risk index (E^i^r) indicates that cadmium has the highest risk level among the studied heavy metals, followed by copper, lead, nickel, chromium, and zinc, which are categorized as having low ecological risks. The risk index (RI) analysis reveals that locations 3 and 6 exhibit moderate ecological risks, while other locations fall into the low ecological risk category. The surface soil (0.5 m depth) poses the highest potential ecological risk. The RI of 174.70 obtained for the dumpsite categorizes it as of moderate ecological risk. These findings emphasize the environmental impact of uncontrolled municipal waste dumping and also highlight the need for routine monitoring of waste disposal sites in developing countries, particularly Nigeria. This is particularly to mitigate potential health impacts on the surrounding community and preserve environmental and public health. Based on the findings, it is therefore recommended that immediate attention and remediation efforts are necessary to mitigate the environmental impact of the Igbatoro dumpsite.

## Supplementary Information

Below is the link to the electronic supplementary material.

Supplementary file1 (XLSX 30 KB)

Supplementary file2 (XLSX 22 KB)

Supplementary file3 (XLSX 23 KB)

## Data Availability

Raw data were generated in the Igbatoro dumpsite, Ondo State, Nigeria. The data from these findings are available from the corresponding author (Folahan O Ayodele) on request.
